# Valgus instability of the elbow: acute and chronic form

**DOI:** 10.1007/s11678-018-0465-1

**Published:** 2018-05-30

**Authors:** Laurent Willemot, Filip R. Hendrikx, Ann-Maria Byrne, Roger P. van Riet

**Affiliations:** 1 Orthopedic Center Antwerp, Antwerp, Belgium; 2 Monica Hospital, Stevenslei 20, 2100 Antwerp, Belgium; 3 University of Antwerp, Antwerp, Belgium; 4 Blackrock Clinic, Dublin, Ireland; 5 Sports Surgery Clinic, Dublin, Ireland

**Keywords:** Elbow, Athletes, Ligaments, Joint instability, Reconstructive surgery, Ellenbogen, Sportler, Ligamente, Gelenkinstabilität, Chirurgische Rekonstruktion

## Abstract

There has been an increase in thrower-specific elbow injuries in recent years. High valgus stresses during the late cocking and acceleration phases of throwing need to be compensated by the flexor pronator muscles as these can exceed the tensile strength of the medial collateral ligament complex. Prevention of injuries is the priority, with a focus on strengthening, reducing throwing frequency, decreasing force, and promoting a technique. The spectrum of thrower injuries ranges from a simple sprain to complete failure of the valgus stabilizing factors. The medial collateral ligament can stretch, leading to posteromedial impingement and radiocapitellar compression forces. This in turn can result in arthrosis and the formation of osteophytes. Ligament failure may eventually occur, making it impossible for the athlete to continue their throwing activities. The outcome of conservative treatment with strengthening, improvement of technique, and relative rest is often disappointing. Direct repair may no longer be possible in these acute-on-chronic injuries and a reconstruction with a tendon graft may be necessary.

## Zusammenfassung

In den letzten Jahren gab es einen Anstieg wurfspezifischer Ellenbogenläsionen. Eine hohe Valgusbelastung während der späten Anspannungs- und Beschleunigungsphasen des Werfens muss durch die Flexoren und Pronatoren ausgeglichen werden, da diese die Zugkraft des medialen kollateralen Bandkomplexes übersteigen können. Der Prävention von Verletzungen wird der Vorzug gegeben, dabei liegt ein Fokus auf der Stärkung, der Verminderung der Wurfhäufigkeit, der Verminderung des Krafteinsatzes und einer ausgefeilten Technik. Werferverletzungen umfassen ein Spektrum von Verletzungen von einer einfachen Zerrung bis zum vollständigen Ausfall der valgusstabilisierenden Faktoren. Das mediale kollaterale Band kann gedehnt werden, was zu einem posteromedialen Impingement und Kompressionskräften zwischen dem Radius und dem Capitulum humeri führt. Dies kann in einer Arthrose und der Bildung von Osteophyten enden. Das Ligament kann schließlich reißen, was es für den Sportler unmöglich macht, seine Wurfsportart weiter auszuführen. Das Ergebnis der konservativen Therapie mit Stärkung, Verbesserung der Technik und relativer Ruhe ist oftmals enttäuschend. Eine direkte Rekonstruktion ist bei diesen chronischen Verletzungen mit akuter Phase ggf. nicht mehr möglich, und u. U. bedarf es einer Rekonstruktion mit einem Sehnenimplantat.

A steady rise in the number of athletes participating in overhead throwing sports has been observed in recent years . This rise has been accompanied by an increased incidence of thrower-specific elbow injuries. Overhead athletes, such as baseball pitchers, javelin throwers, and handball players, are at risk of developing medial elbow symptoms due to the high valgus stresses generated during throwing. Similarly, power grip (racket) sports, gymnastics, and weight-lifting are associated with medial elbow injuries.

A combination of tensile forces at the medial stabilizing structures, lateral compartment compression, and posterior shear forces may lead to valgus instability. Chronic problems are associated with repetitive motion and overuse, yet acute and acute-on-chronic injuries also occur. A thorough understanding of the functional anatomy of the elbow as well as the biomechanics of throwing is essential when treating this unique type of sports injury.

This review discusses the topic of acute and chronic valgus instability of the elbow covering the relevant anatomy, biomechanics, clinical examination, imaging modalities, and treatment options for this condition.

## Anatomy and biomechanics

The elbow joint functions as a modified hinge. Both static and dynamic stabilizers help safeguard elbow stability throughout range of motion (ROM) and under external destabilizing forces.

The ulnohumeral articulation acts as the primary stabilizer at the end-ROM between 0° and 20° of extension and 120° 140° of flexion. Both static and dynamic structures are required to stabilize the elbow in the midrange of 100° [34, 35].

Both static and dynamic structures are required to stabilize the elbow

**Fig. 1:**
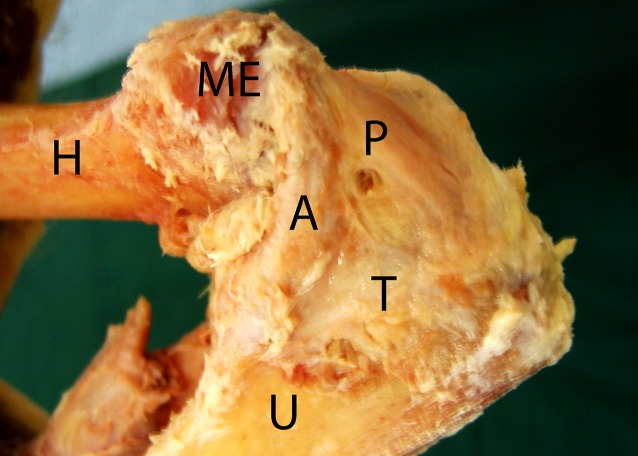
Cadaveric dissection of the medial side of the elbow. Three parts of the medial collateral ligament (MCL) complex are distinguished. *H* humerus, *U* ulna, *ME* medial epicondyle, *A* anterior band of the MCL, *P* posterior band, *T* transverse band. (Courtesy of the MoRe Foundation)

In full extension, the osseous constraints, the medial collateral ligament (MCL), and the anterior capsule form the mainstay of valgus stability [18], while the MCL serves as a primary restraint between 30°and 110° of flexion [24]. The MCL is composed of three distinct bands: the anterior, posterior, and transverse bands (Fig. 1). The anterior band, which originates on the under-surface of the medial epicondyle and inserts on the sublime tubercle of the proximal ulna, acts as the primary stabilizer to valgus stress in flexion between 30° and 120° [24]. Because of the anterior insertion, the anterior band is tighter in extension and in early stages of elbow flexion when compared with the posterior band. Since the posterior band inserts posterior to the anterior band, it tightens more in further stages of flexion. The posterior bundle alone contributes little to overall stability; however, at 30° of flexion it acts as a secondary stabilizer, becoming functionally more important between 60° and full flexion [39]. The posterior band may play an important role in posteromedial instability of the elbow [36]. The transverse band is believed to regulate the relationship between the anterior and posterior bands but has been shown to be of little significance in resisting valgus stress when sectioned in a cadaveric study [39].

The radial head serves as a secondary stabilizer to valgus stress [24] contributing up to 30% of medial stability with an intact anterior band of the MCL [14, 24, 28, 34, 35].

In the case of MCL insufficiency, the radial head becomes the primary stabilizer against valgus instability [24, 40].

The dynamic stabilizers of the flexor–pronator muscle group counteract the valgus stress forces of the throwing motion and are of vital importance in prevention and rehabilitation strategies [3]. Unfortunately, these muscles cannot fully compensate for a torn MCL complex and electromyography (EMG) studies have shown that, paradoxically, activity is decreased in the presence of an MCL injury. This reflects the inability of the flexor–pronator group to sufficiently compensate for the loss of valgus stability after MCL rupture in overhead athletes [15].

## Mechanisms of injury

Most cases of symptomatic chronic valgus instability occur as a result of repetitive trauma to the medial elbow stabilizers in overhead athletes. Much of the research into valgus instability has focused on throwing injuries in baseball pitchers. The overhead throwing motion in baseball has been divided into six stages:

1. Windup
2. Early cocking
3. Late cocking
4. Acceleration
5. Deceleration
6. Follow-through [13]

**Fig. 2:**
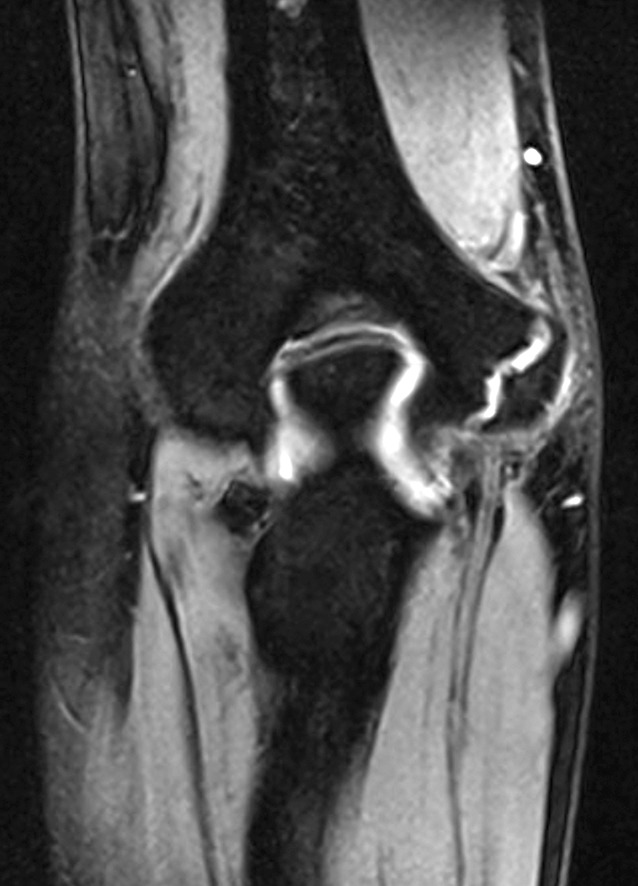
Magnetic resonance image of a medial epicondyle apophysitis in a skeletally immature athlete. (Courtesy of the MoRe Foundation)

During the late cocking and acceleration phases, the MCL complex of the elbow experiences valgus stresses reaching up to 64 Nm, exceeding the ultimate tensile strength of the anterior bundle of the MCL [2, 42]. Contraction of the flexor–pronator group mitigates the remaining force [30]. However, if the muscular compensation fails, injury to the MCL may occur. Furthermore, due to the simultaneous elbow extension that occurs during the throwing motion, bending moments arise within the anterior MCL bundle, which can lead to destructive shear forces between the ligament’s fibers. Repetitive microtrauma to the ligamentous complex can result in stretching and attenuation of the MCL and can eventually lead to a full acute-on-chronic rupture. These acute-on-chronic ruptures are therefore often intraligamentous tears, whereas acute traumatic injuries usually result in an avulsion of the ligament from its humeral insertion. Secondary stabilizers and neurovascular structures on the medial side of the elbow may also be injured as a result of valgus laxity, resulting in flexor–pronator mass tendinopathy, ulnar neuritis, or medial epicondyle apophysitis in the skeletally immature patient (Fig. 2).

**Fig. 3:**
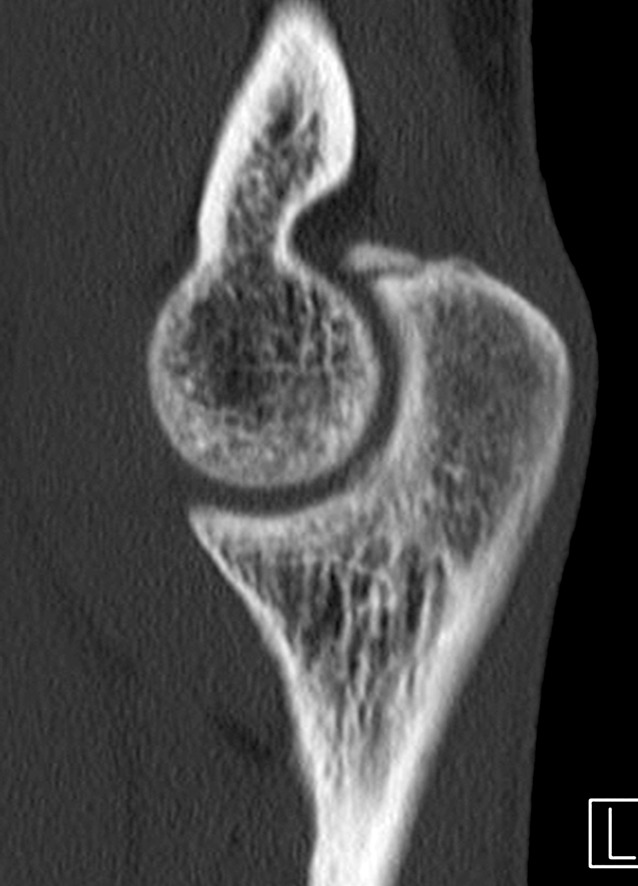
Computed tomography scan of the elbow showing a fractured osteophyte at the tip of the olecranon. (Courtesy of MoRe Foundation)

At the end of the throwing motion, in the so-called follow-through stage, shear forces on the posterior compartment may produce posteromedial olecranon impingement in extension, with a corresponding lesion in the olecranon fossa (Fig. 3). Moreover, valgus stresses on the medial side are typically accompanied by overload in the lateral compartment of the elbow. This can lead to abnormally high compressive forces across the radiocapitellar articulation. Such forces may damage the cartilage, resulting in chondromalacia, osteophytes, and loose bodies. A combination of excessive medial tensile forces together with lateral compartment compression and posterior shear forces during throwing motion is termed valgus extension overload syndrome (VEOS) [5].

Untreated valgus elbow instability can lead to early joint degeneration

Less frequently, valgus instability occurs as the result of unrecognized or neglected trauma or after failed treatment for acute elbow instability. In rare occasions, valgus instability is associated with connective tissue disease, rheumatic conditions, or neurological impairment.

Unrecognized or untreated valgus elbow instability can lead to early joint degeneration due to abnormal joint kinematics creating high-stress areas and cartilage destruction. Moreover, recurrence and symptoms of elbow instability may influence the performance and earning potential of athletes.

## Diagnosis

### History

In the assessment of valgus elbow instability, valuable information can be gained from a thorough patient history. Details of the exact moment of the injury, the events leading up to and following it, prior injuries, changes in training regimen, racket tension and grip, and professional occupation must be obtained. In acute cases, the patient may recall a sudden tangible or audible “pop” accompanied by acute pain and a limitation in ROM [27]. More often, patients with chronic injuries will report insidious but gradually worsening or fluctuating symptoms, specifically during a particular causative motion such as throwing. Athletes may also report progressive loss of ball control and throwing performance. The pain usually reaches maximal intensity during the late cocking and early acceleration phases, but with VEOS, athletes may also report posteromedial pain during the deceleration phase caused by posterior osteophyte impingement. Ulnar nerve symptoms at rest or during the provoking motion should also be recorded.

### Clinical examination

**Fig. 4:**
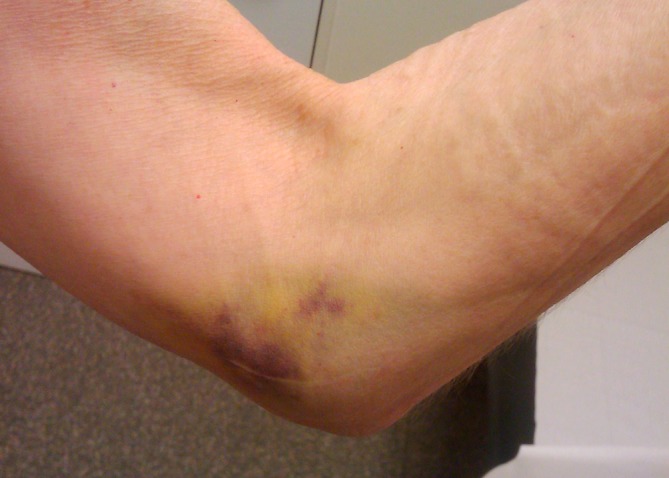
Clinical photograph of the medial side of the elbow following an elbow dislocation. Ecchymosis is indicative of a medial collateral ligament injury. (Courtesy of the MoRe Foundation)

Clinical examination typically starts with inspection of the joint in resting position. Ecchymosis may be present in the case of elbow dislocation (Fig. 4). Palpable fullness of the soft spot is a sign of intra-articular effusion. In the case of acute effusion, the patient will hold the elbow at a flexion angle of 70°, to accommodate the increased intracapsular volume. The carrying angle, between the humerus and forearm, may be higher than the average 11° and 13° in men and women, respectively, as a result of repetitive valgus stretch and MCL elongation [5]. Palpation of the bony structures is paramount during the examination. Point tenderness at the MCL insertion on the sublime tubercle, which is a frequent site of pain in valgus injuries, is indicative of valgus stress injury. Medial epicondyle pain in skeletally immature patients may indicate an avulsion injury after elbow trauma. Active and passive ROM should be assessed with special attention to pain, crepitus, and locking or loss of motion. A hard extension block may indicate a loose body or posterior osteophyte.

#### Specific tests

The patency of the anterior bundle of the MCL is best evaluated by different valgus stress tests. Valgus stress is applied in various angles of flexion. To examine the MCL, the elbow is flexed to 20°–30° to unlock the joint. The examiner stabilizes the right humerus with the left hand just above the elbow and applies a valgus moment with the right hand on the patient’s forearm. The contralateral elbow is then tested for comparison.

With the “milking maneuver” [41], valgus stress can be applied to the anterior bundle of the MCL by grasping the supine patient’s thumb on the affected side, with the patient’s arm in 90° shoulder abduction and 90° elbow flexion. A valgus stress is then applied by pulling down on the thumb, as one would pull down when milking a cow. Reproduction of pain indicates a positive test result.

In the “moving valgus stress” test, the patient stands with the shoulder abducted at 90°. The shoulder is maximally externally rotated. The elbow is then rapidly extended from maximal flexion to 30° under a constant valgus force applied to the patient’s thumb [27]. For a positive result, two conditions must be satisfied: the pain elicited must be similar to that during the causative motion; and maximal pain must occur during the position of late cocking (120° elbow flexion) and early acceleration (30° elbow flexion).

Particular attention should be paid to the ulnar nerve in cases of valgus instability. The elbow should be evaluated for a Tinel sign at the cubital tunnel, and nerve stability should be assessed when moving from extension to flexion.

### Imaging

While the diagnosis of medial elbow instability is primarily based on the patient history and clinical findings, imaging studies may reveal unsuspected concomitant lesions.

**Fig. 5:**
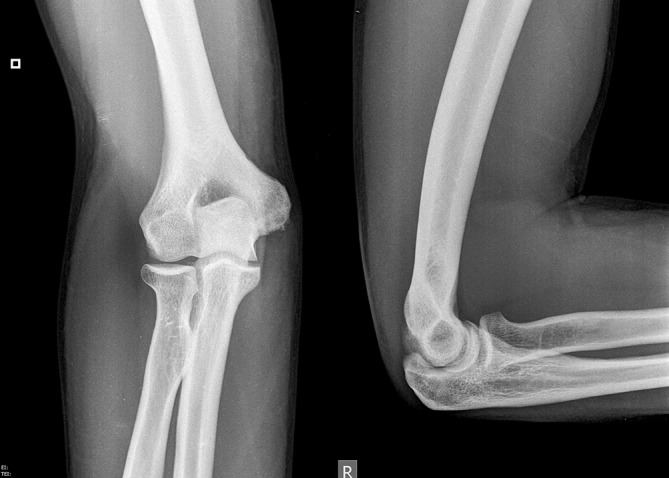
Plain radiographic anteroposterior and lateral views of the elbow, showing calcification of the medial epicondyle as an indirect sign of chronic instability. (Courtesy of the MoRe Foundation)

Plain static radiographs may reveal calcifications of the MCL, indicating chronic valgus instability (Fig. 5). Loose bodies, osteophytes, and radiocapitellar pathology may also be seen on standard radiographs.

**Fig. 6:**
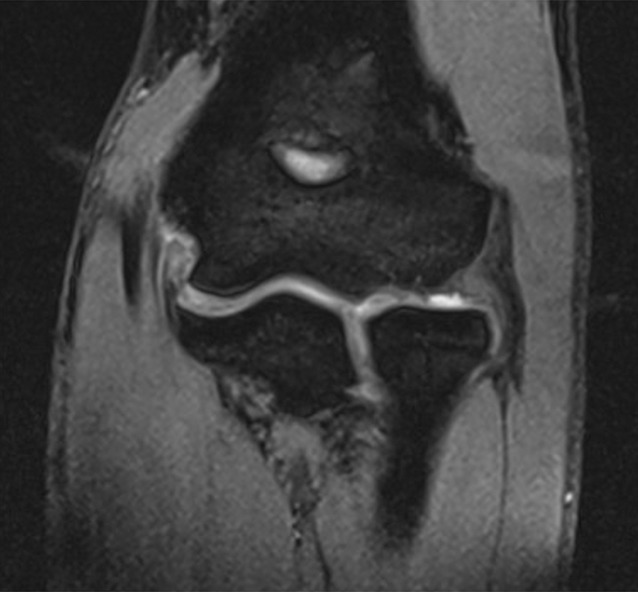
Magnetic resonance image of the elbow, showing a full-thickness tear of the medial collateral ligament. (Courtesy of the MoRe Foundation)

**Fig. 7:**
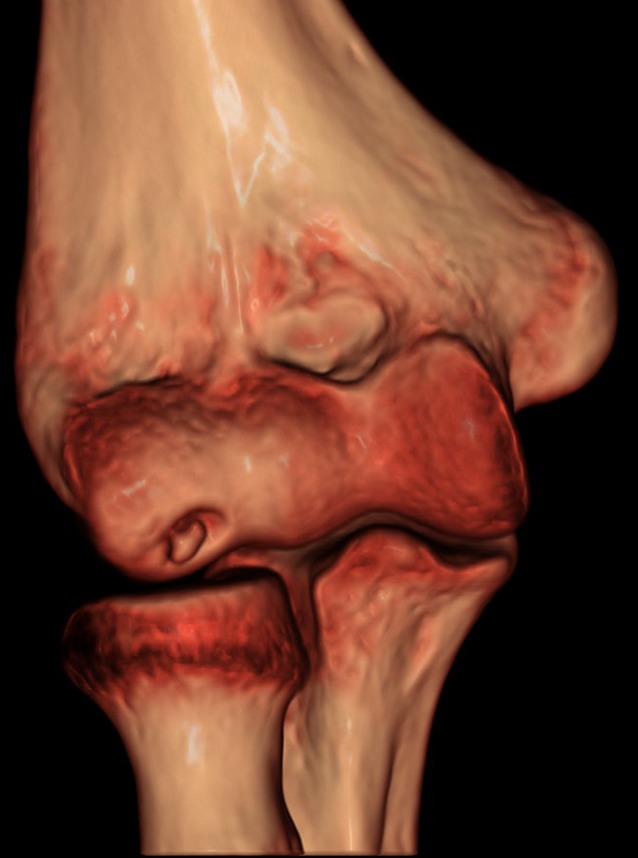
Three-dimensional computed tomography scan showing sequelae of osteochondritis dissecans caused by valgus extension overload syndrome prevalent in young throwing athletes. (Courtesy of the MoRe Foundation)

Valgus stress radiographs can be helpful in cases with equivocal clinical findings. A medial joint line opening of more than 3 mm is considered consistent with valgus instability [19, 38]. Advanced imaging modalities such as computed tomography (CT) and magnetic resonance imaging (MRI) can be valuable tools in the diagnosis of valgus instability (Fig. 6). CT is most helpful in cases with associated bone lesions such as osteochondritis dissecans, fractures, osteophyte formation, and loose bodies (Fig. 7). Contrast-enhanced CT scans allow for the visualization of the medial ligamentous structures. However, we prefer the use of MRI for soft tissue evaluation. MRI can aid in the detection of MCL tears, osteochondral injuries, olecranon osteophytes, loose bodies, and sites of neurologic compression [11, 23, 26]. The addition of contrast arthrography to MRI improves visualization of partial under-surface MCL tears [17]. Saline-enhanced MRI facilitates the evaluation of leakage through the MCL, increasing the sensitivity of the examination from 57 to 92% [25].

## Treatment

### Nonoperative management

Conservative treatment consists of a rehabilitation program after a period of rest and adequate pain control. Immediate mobilization is important in the prevention of stiffness and has been shown not to increase the risk of recurrent instability [22]. A dynamic brace can be applied for comfort and for reducing valgus stress on the elbow, with a stepwise increase to full extension. The program should include strengthening of the wrist flexor–extensor [16] and flexor–pronator muscle groups together with retraining of throwing mechanics in the case of overhead athletes. Optimizing the technique is most important in patients with VEOS without an MCL tear.

Immediate mobilization is important for preventing stiffness

The risk of recurrent symptoms is extremely high if the underlying cause is not corrected, even when conservative treatment is successful initially. Rettig et al. noted that only 42% of baseball pitchers returned to their pre-injury sports level after an average time of 24.5 weeks [32]. Chronicity of the injury and patient age did not seem to influence the prognosis of conservative treatment in their study.

### Operative management

Indications for surgical MCL reconstruction require a confirmatory history, physical examination, and imaging studies. High-demand patients with a diagnosis of MCL insufficiency for whom nonoperative treatment has failed are candidates for surgical reconstruction [21].

**Fig. 8:**
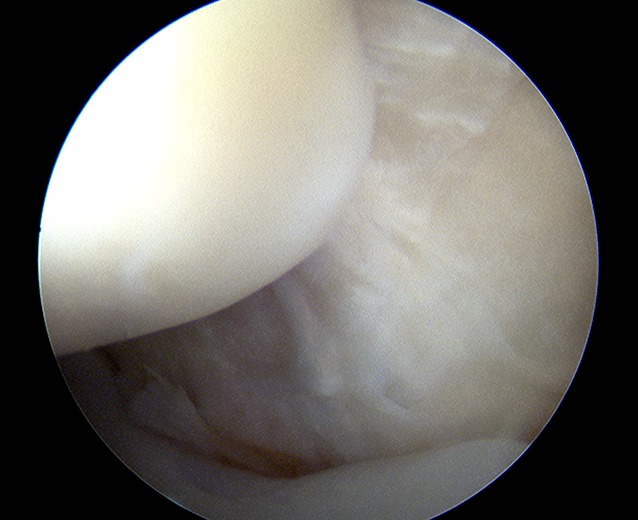
Arthroscopic view of the posteromedial elbow. There is significant opening with valgus stress, indicating a complete tear of the medial collateral ligament complex. (Courtesy of the MoRe Foundation)

Some authors advocate elbow arthroscopy before formal MCL repair for two reasons: (a) arthroscopy may play a role in the diagnosis of valgus instability (Fig. 8)—a gap of more than 1 mm at the medial ulnohumeral joint line is indicative of valgus instability [12]; and (b) arthroscopy may be able to address potential concomitant lesions such as posteromedial impingement and loose body removal.

Early surgery may be indicated in cases of acute valgus instability

Early surgery may be indicated in the case of acute valgus instability, confirmed on imaging studies, in a high-demand patient. Patients presenting with a partial tear and the absence of bony abnormalities, yet who are resistant to conservative treatment, present a decision-making challenge.

**Fig. 9:**
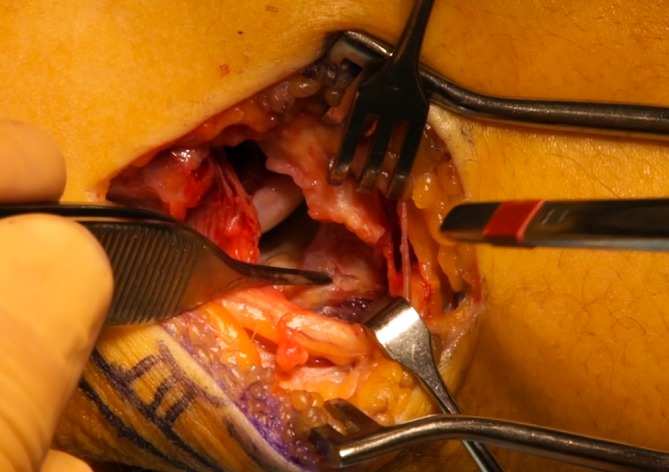
Intraoperative view of the elbow showing complete avulsion of the medial collateral ligament. (Courtesy of the MoRe Foundation)

**Fig. 10:**
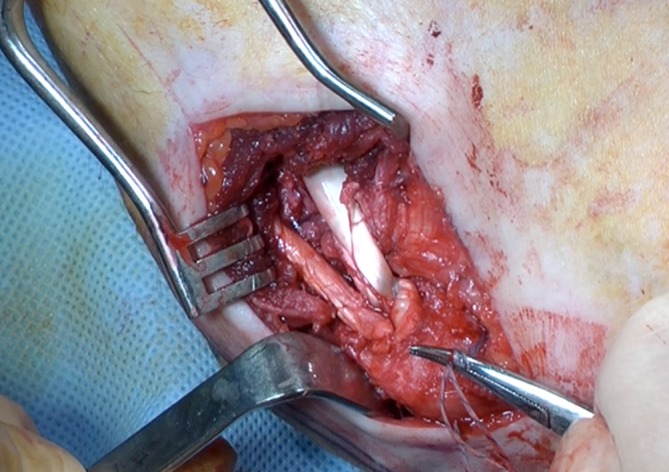
Medial collateral ligament reconstruction using an extensor hallucis longus graft. The graft is fixed through a bone tunnel in the ulna and a docking technique is used to fix the graft in the humerus. (Courtesy of the MoRe Foundation)

Direct repair of the ruptured MCL is only indicated in cases of acute avulsion from either the humeral origin or the coronoid insertion (Fig. 9; [6, 7, 19]). The direct repair may be reinforced with a tendon graft via a hybrid technique. Most MCL reconstruction techniques involve a free tendon graft, typically placed in bone tunnels through the humerus and ulna (Fig. 10). Graft options that have been previously described include autologous and allograft palmaris longus tendon, plantaris tendon, hamstring tendons, and strips of Achilles or triceps tendon. Jobe and colleagues [19, 37] described the original MCL reconstruction technique consisting of (a) tendinous transection and reflection of the flexor–pronator mass, (b) submuscular transposition of the ulnar nerve, and (c) creation of humeral tunnels that penetrate the posterior humeral cortex. Although this technique was successful, it was technically demanding and associated with a high complication rate, most often related to ulnar nerve problems [19, 31]. Since then, the technique has been modified. A muscle-splitting approach has been developed to avoid detachment of the flexor–pronator mass and tunnels are drilled anteriorly on the humeral epicondyle to avoid the risk of ulnar nerve injury. The number of bone tunnels has also been reduced to facilitate graft tensioning and avoid the risk of iatrogenic fracture [1, 2, 33].

Several methods of graft fixation have been described, including transosseous figure-of-eight reconstruction, docking technique, hybrid interference screw fixation, and EndoButton fixation [2, 4, 33].

### Outcome

Recent studies have demonstrated a 93% success rate [2, 8, 9, 29, 38] with current ligament reconstruction techniques. A high rate of return to play (RTP) in elite athletes and a high rate of return to a pre-injury or higher level have been reported [4, 20, 33]. Azar et al. [4] noted an 81% return to pre-injury level, Rohrbough et al. [33] found a 92% RTP, and Erickson et al. [10] found that 83% of patients were able to return to the same level.

## Summary

The incidence of medial-sided elbow injuries has risen in recent years owing to the increased participation in overhead throwing sports. The spectrum of injuries comprises acute strain or rupture of the MCL to chronic valgus overload syndrome, leading to arthritis and MCL insufficiency. This common pattern of symptoms is referred to as valgus extension overload syndrome. The diagnosis is mainly clinical with several special tests. Radiographic imaging, CT, and MRI may be used to confirm the clinical diagnosis but are most helpful in diagnosing associated pathology such as cartilage lesions, osteophytes, or loose bodies.

Treatment options depend on the sportive and professional demands of the patient. Management of medial elbow symptoms in nonthrowing athletes and low-demand patients may be nonoperative. Conservative treatment will initially include rest and anti-inflammatory measures followed by strengthening exercises and progressive valgus loading of the elbow. A thorough evaluation of the causative motion and the athletic technique is imperative for successful conservative treatment. Conservative treatment may be disappointing in the face of degenerative changes in the elbow joint. If present, loose bodies or osteophytes should be removed arthroscopically. Elbow arthroscopy provides the added benefit of direct MCL inspection. A medial opening of the joint space by more than 1 mm is indicative of insufficiency.

Direct repair of the acutely avulsed MCL may be indicated for selected patients; however, as the quality of the ligament is usually low from chronic overuse, a reconstruction or hybrid technique is typically warranted. Surgical reconstruction of the MCL is indicated in high-demand patients with complete MCL tears or those with partial tears for whom rehabilitation has failed. Jobe’s original MCL reconstruction technique has seen technical modifications over the past 30 years. A successful outcome after MCL reconstruction hinges on decreased dissection of the flexor–pronator mass, minimal handling of the ulnar nerve, and recognition and treatment of concomitant medial and intra-articular elbow pathology.

## Practical conclusion

- Thrower-specific elbow injuries have increased in recent years.
- Prevention of injuries is the priority, with a focus on strengthening, reducing throwing frequency, decreasing force, and optimizing the athlete’s technique.
- Thrower injuries range from a simple sprain to complete failure of the valgus stabilizing factors. The MCL can stretch, leading to posteromedial impingement and radiocapitellar compression forces that can result in arthrosis and osteophyte formation. Ligament failure may eventually occur, making it impossible for athletes to continue with their throwing activities.
- The outcome of conservative treatment is often disappointing. Direct repair may no longer be possible in these acute-on-chronic injuries and reconstruction with a tendon graft may be necessary.
- A successful outcome after MCL reconstruction depends on decreased dissection of the flexor–pronator mass, minimal handling of the ulnar nerve, and treatment of concomitant medial and intra-articular elbow pathology.
